# Sarcopenia y albúmina sanguínea: revisión sistemática con metaanálisis

**DOI:** 10.7705/biomedica.5765

**Published:** 2021-09-22

**Authors:** Jack Roberto Silva-Fhon, Violeta Magdalena Rojas-Huayta, Juan Pablo Aparco-Balboa, Bernardo Céspedes-Panduro, Rosalina Aparecida Partezani-Rodrigues

**Affiliations:** 1 Escola de Enfermagem, Universidade de São Paulo, São Paulo, Brasil Universidade de São Paulo Universidade de São Paulo São Paulo Brazil; 2 Centro Brasileiro para o Cuidado à Saúde Baseado em Evidências: Centro de Evidência do JBI, São Paulo, Brasil Centro de Evidência do JBI São Paulo Brasil; 3 Núcleo de Investigación en Alimentación y Nutrición Pública, Escuela de Nutrición, Facultad de Medicina, Universidad Nacional Mayor de San Marcos, Lima, Perú Universidade São Marcos Facultad de Medicina Universidad Nacional Mayor de San Marcos Lima Brazil; 4 Escuela de Estadística, Facultad de Matemáticas, Universidad Nacional Mayor de San Marcos, Lima, Perú Universidade São Marcos Escuela de Estadística Facultad de Matemáticas Universidad Nacional Mayor de San Marcos Lima Brazil; 5 Facultad de Estudios Generales, Universidad Privada del Norte, Lima, Perú Universidad Privada del Norte Facultad de Estudios Generales Universidad Privada del Norte Lima Peru; 6 Escola de Enfermagem de Ribeirão Preto, Universidade de São Paulo, Ribeirão Preto, Brasil Universidade de São Paulo Escola de Enfermagem de Ribeirão Preto Universidade de São Paulo Ribeirão Preto Brazil

**Keywords:** sarcopenia, albúminas, anciano, biomarcadores, revisión sistemática, metaanálisis, envejecimiento, Sarcopenia, albumins, aged, biomarkers, systematic review, meta-analysis, aging

## Abstract

La sarcopenia se caracteriza por la pérdida de musculatura durante el envejecimiento, lo que puede traer consecuencias para la salud. Se detecta de diversas formas, una de ellas, el uso de biomarcadores sanguíneos como la albúmina, aunque todavía no se ha establecido dicha asociación de forma definitiva.

Esta revisión sistemática y metaanálisis resume el conocimiento sobre la asociación entre sarcopenia y albúmina sérica en los adultos mayores, centrada en la etiología y los factores de riesgo. La revisión se hizo utilizando el programa del *Joanna Briggs Institute* y la búsqueda incluyó las bases de datos Medline, Embase, CINAHL, y LILACS; la búsqueda manual estuvo a cargo de dos revisores de forma independiente. Para el metaanálisis, se utilizó el programa EpiDat, versión 3.1; las diferencias de medias en los puntajes de albúmina desagregados por sarcopenia se analizaron mediante el modelo de efectos aleatorios. El grado de heterogeneidad se evaluó con la prueba Q de DerSimonian y Laird. Se analizaron 630 artículos, de los cuales 14 se incluyeron en la revisión. En el metaanálisis, se evidenciaron mayores cantidades de albúmina sanguínea en los adultos mayores que no presentaron sarcopenia frente a los que sí, una diferencia con significación estadística. Aunque hay estudios en los que se explora la asociación entre albúmina y sarcopenia, persiste la necesidad de evaluar la asociación entre los marcadores biológicos, comparándolos entre sí para determinar cuáles pueden utilizarse en la detección de sarcopenia en el adulto mayor.

## Abstract (en)

Sarcopenia is characterized by loss of muscle mass during aging, which can have consequences for the individuals' health. There are many ways to detect it, among them, with the use of blood biomarkers such as albumin, although the association between the two has not been confirmed yet.

This review synthesizes the knowledge on the association between sarcopenia and serum albumin among elderly individuals through a systematic review and meta-analysis focused on the etiology and risk factors. We used the Joanna Briggs Institute software for the review and conducted a search in MEDLINE, Embase, CINAHL, and LILACS databases while two reviewers conducted an independent manual search. EpiDat, version 3.1 was used for the meta-analysis; mean differences with the albumin scores disaggregated by sarcopenia were analyzed by the random-effects model. The degree of heterogeneity was assessed with the DerSimonian and Laird Q test.

We analyzed 630 articles and finally included 14 in the review. Higher blood albumin levels were found in the meta-analysis, which was statistically significant among the elderly adults who did not present sarcopenia compared to those who did. Although there are studies exploring the association between albumin and sarcopenia, there is a need to continue evaluating its association with biological markers and comparing them to verify which can be used to detect sarcopenia among the elderly.

El informe de las Naciones Unidas sobre envejecimiento de la población mundial refiere que, en las próximas tres décadas, el número global de personas mayores de 65 años se duplicará, llegando a más de 2.000 millones para el 2050 ^1^. El acentuado incremento de la población de adultos mayores es una oportunidad para brindarles todas las condiciones necesarias para que tengan un envejecimiento saludable hasta donde lo permitan los cambios estructurales y funcionales propios del proceso de maduración biológica ^2^.

## Sarcopenia y albúmina en el adulto mayor

La pérdida progresiva de funcionalidad y el aumento de la mortalidad representan procesos fundamentales del envejecimiento biológico intrínsecos en la mayoría de los sistemas celulares. El deterioro muscular durante el envejecimiento se produce por dos mecanismos subyacentes que regulan la disminución de la masa y la función muscular: atrofia y pérdida de fibra muscular, también llamada hipoplasia ^3^^,^^4^.

Uno de los síndromes relacionados con la atrofia muscular es la sarcopenia. Según el último consenso europeo, esta se considera una enfermedad muscular en la que ocurre una disminución de la fuerza y la masa muscular, lo cual determina el inicio de la investigación diagnóstica. Por otro lado, la disminución de la actividad física es una medida de la gravedad de la sarcopenia en el adulto mayor ^5^. Se estima que la prevalencia de sarcopenia en Suramérica es de alrededor de 13,9 % (IC_95%_ 12,0-15,8 %); además, es significativamente mayor entre los adultos mayores con baja escolaridad y nivel socioeconómico, que no tienen compañero, son fumadores, sedentarios y tienen bajo índice de masa corporal ^6^.

Por otra parte, la pérdida significativa de masa muscular en personas de edad avanzada se ha asociado con bajos niveles de albúmina sérica, proteína que se considera un indicador clave del estado nutricional ^7^^,^^8^. Sin embargo, la concentración de albúmina se ve afectada también por procesos inflamatorios e infecciosos, entre otros, por lo que aún no hay consenso sobre sus límites óptimos y los rangos de referencia de sus valores séricos para la evaluación del estado nutricional en el adulto mayor ^8^^,^^9^.

Considerando que el estado nutricional deficiente y la sarcopenia pueden superponerse, la albúmina sérica podría considerarse como un biomarcador para la detección de este síndrome, ya que no se dispone de otros que sean de fácil aplicación y bajo costo, y garanticen una evaluación oportuna y una pronta intervención ^9^. Sin embargo, todavía no hay evidencia concluyente para considerar la albúmina como biomarcador de la sarcopenia.

Dado el importante incremento de la población de adultos mayores y la significativa prevalencia de sarcopenia en este grupo poblacional, es imprescindible evaluar marcadores biológicos que permitan diagnosticarla de manera rápida y sencilla, con la finalidad de prevenir este síndrome, mejorar el estado nutricional, y promover un envejecimiento activo y saludable.

En ese sentido, los integrantes de los grupos de investigación *Prática Pedagógica no Ensino Superior de Enfermagem e no Cuidado à Saúde do Adulto e Idoso* de la *Universidade de São Paulo* y el Núcleo de Investigación en Alimentación y Nutrición Publica de la Universidad Nacional Mayor de San Marcos, desarrollaron el presente estudio para contribuir a que los profesionales de la salud comprendan la importancia de valorar y detectar la sarcopenia asociada con biomarcadores sanguíneos como la albúmina sérica. En ese marco, se resumió la información sobre dicha asociación en los adultos mayores por medio de una revisión sistemática con metaanálisis.

## Desarrollo de la revisión

En la revisión sistemática con metaanálisis, se utilizaron la metodología y las pautas del Joanna Briggs Institute, cuyo objetivo es proporcionar una síntesis exhaustiva e imparcial de estudios relevantes en un solo documento mediante métodos rigurosos y transparentes en la búsqueda de evidencia relevante para la pregunta de estudio ^10^.

En el estudio se plantearon las siguientes etapas: 1) título de la revisión; 2) objetivo y pregunta del estudio; 3) introducción (antecedentes); 4) criterios de inclusión; 5) métodos (estrategia de búsqueda, evaluación crítica, selección de los estudios y síntesis de los datos); 6) resultados; 7) discusión, y 8) conclusión y recomendaciones ^11^.

En el título, se recogieron los elementos principales de la pregunta en estudio y se aseguró que se relacionara con el objetivo y los criterios de inclusión. En la formulación del objetivo y la pregunta de la revisión, se utilizó la estrategia PEO, en la cual P es la población (adultos mayores), E es la exposición de interés (estado nutricional determinado con base en la detección de albúmina sanguínea) y O es el resultado (sarcopenia). Esta estrategia facilitó la formulación de la siguiente pregunta: ¿Qué conocimiento está disponible en la literatura médica sobre la asociación entre la sarcopenia y el estado nutricional medido con base en la albúmina sanguínea en los adultos mayores?

Los criterios de inclusión fueron los siguientes: estudios con participantes de 60 años de edad o más, independientemente del sexo, el origen étnico, el estado social y los diferentes entornos (hospital, casa o casas de reposo); estudios longitudinales en los idiomas portugués, inglés y español, sin límite de fecha de publicación. Los criterios de exclusión, por otra parte, fueron: estudios de revisión; tesis; capítulos de libros; informes técnicos, resúmenes publicados y cartas al editor.

La búsqueda de estudios se hizo el 9 de julio de 2020, con una actualización el 12 de abril de 2021, en las bases de datos *National Center for Biotechnology Information* (NCBI/PubMed), *Cumulative Index to Nursing and Allied Health Literature* (CINAHL), *Excerpta Medical Database* (EMBASE) y *Latin-American and Caribbean Center on Health Sciences Information* (LILACS), empleando los descriptores y palabras clave presentadas en el cuadro 1. Además, se utilizaron los operadores booleanos OR y AND para obtener combinaciones sumatorias y restrictivas, respectivamente.

**Cuadro 1:** Estrategia de búsqueda de los artículos en las diferentes bases de datos, y descriptores y palabras clave empleadas. Periodo: julio de 2021

	P	E	O
Inglés (PubMed - MeSH)	Aged AND elderly OR older OR older people OR ancient OR elderly people OR very elderly	Albumins AND Hypoalbuminemia OR Albumin OR serum albumin OR Albuminoid OR hypoalbuminemia	Sarcopenia AND sarcopenias
Portugues (DeCS)	Idoso AND pessoa idosa OR pessoa de idade OR pessoas idosas OR pessoas de idade OR populacao idosa OR centenarios OR velhissimos	Albuminas AND Albumina OR Albumina plasmatica OR albumina do plasma OR Albumina sérica humana	Sarcopenia
Espanol (DeCS)	Anciano AND adulto mayor OR persona de edad OR viejo OR poblacion anciana OR personas adultos mayores	Albuminas AND albumina plasmatica OR albumina plasmática OR Albumina serica humana	Sarcopenia

En la recopilación de la información, se siguieron las recomendaciones del *Preferred Reporting Items for Systematic Reviews and Meta-Analyses* (PRISMA), con el fin de aumentar el número de publicaciones, y proporcionar información más completa y transparente ^12^, con lo que se encontraron 630 artículos. Posteriormente, se eliminaron los artículos duplicados y dos revisores independientes cegados leyeron los títulos y los resúmenes.

Después de esta selección, un tercer revisor analizó y decidió, conjuntamente con los revisores, la inclusión o exclusión de cada artículo, especialmente cuando existía algún conflicto sobre la decisión de incluir el artículo, con lo que se totalizaron 14 para la revisión. Después de la selección con el tercer revisor, se hizo una búsqueda manual a partir de las referencias de los estudios seleccionados.

Para la extracción de los datos de los estudios, se creó un instrumento con detalles específicos relacionados con el tema y el objetivo de la revisión, tales como autor(es), año de publicación, tipo de periódico, idioma, país, título, objetivos, población, contexto, tipos de estudio y método, tamaño de la muestra, enfoque de la albúmina sanguínea, prevalencia de sarcopenia y resultados de los análisis realizados. La información se agrupó en cuadros según la presentación de las medidas de asociación entre las variables estudiadas. La extracción de los datos tiene la finalidad de caracterizar el estudio publicado en cuanto a sus aspectos generales y el método utilizado en la investigación, así como a sus respectivos resultados.

Los resultados se presentaron en forma de síntesis narrativa, caracterizada por el análisis de los datos cuantitativos y, una vez expuestos, la discusión, las conclusiones y las recomendaciones se presentaron en la parte final del estudio.

La calidad metodológica de los estudios seleccionados para la presente revisión se garantizó con dos evaluadores que utilizaron la herramienta *Methodological Index for Non-Randomized Studies* (MINORS) ^13^, compuesta por ocho ítems: objetivo claramente establecido; inclusión de pacientes consecutivos; recolección prospectiva de datos; resultados adecuados al objetivo del estudio; evaluación imparcial de los resultados del estudio; período de seguimiento adecuado para los fines del estudio; pérdida en el seguimiento inferior al 5 %, y cálculo prospectivo del tamaño de la muestra. Cada dominio tiene un puntaje de cero a dos y uno global de cero a 16 puntos ^13^.

Para el análisis estadístico, se utilizó el programa EpiDat, versión 3.1; se hizo un metaanálisis para las diferencias de medias con los puntajes de albúmina desagregados por sarcopenia mediante el modelo de efectos aleatorios, dada la heterogeneidad de los resultados individuales. El grado de heterogeneidad se evaluó con la prueba Q de DerSimonian y Laird, en tanto que, con el gráfico de Galbraith, se valoró el sesgo de publicación utilizando un gráfico de embudo y el valor estadístico de Begg. La sensibilidad se analizó con el método gráfico de influencias, así como el metaanálisis acumulado, para evidenciar los cambios en el puntaje global por la adición de cada estudio individual.

Además, se utilizó un diagrama de bosque para el resultado total del metaanálisis, con el fin de evidenciar las diferencias en cada estudio y globalmente, con sus respectivos intervalos de confianza. En todos los análisis, la significación estadística se estableció en p<0,05.

## Selección de los artículos incluidos en la revisión

**Figura 1:**
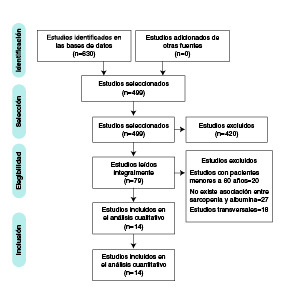
Diagrama de flujo PRISMA para la selección de los artículos.

De los 630 artículos encontrados en las cuatro bases de datos, 131 eran duplicados, con lo que quedaron 499 para la lectura de los títulos y resúmenes. De estos, se excluyeron 420 por no cumplir con los criterios de inclusión y, después de su evaluación, se seleccionaron 79 artículos para su lectura integral. Posteriormente, se excluyeron 65 artículos, lo que resultó en 14 estudios incluidos (figura 1).

Los años de publicación de los 14 estudios analizados (cuadro 2) fueron del 2013 al 2020: cinco en el 2016 (20-24), tres en el 2018 (16-18), dos en el 2013 (26,27) y uno en el 2015 (25), uno en el 2017 (19), otro en el 2019 (15) y otro en el 2020 ^14^. En cuanto al idioma, todos los estudios fueron publicados en inglés. Con respecto al país, se encontró que 11 se realizaron en China, Japón o Corea, y tres, en el Reino Unido e Italia.

**Cuadro 2:** Características de los estudios incluidos en la revisión sistemática según autores, población, variables demográficas, método, sarcopenia y albúmina.

Autor/año	Población	Edad (Media; DE)	Sexo (%)	Método de estudio	Método para identificar la sarcopenia	Prevalencia de sarcopenia	Valor de la albúmina (DE) g/L
Abbas, *et al.*, 2020 ^14^	462	Sarcopenia 82,23 No sarcopenia 80,93	Hombres (29,2) Mujeres (70,89	Longitudinal prospectivo	European Working Group on Sarcopenia in Older People	33,5 %	Sarcopenia 3,38 (0,05)
Gong, *et al.*, 2019 ^15^	168	83,34 (7,32)	Hombres 99 (58,93); Mujeres 69 (41,07)	Longitudinal retrospectivo	Asian Working Group for Sarcopenia	72,02 %	Sarcopenia 32,64 (3,41) No sarcopenia 38,60 (2,03)
D’Alessandro, *et al.*, 2018 ^16^	80	73,7 (7,2)	_____	Longitudinal prospectivo	European Working Group on Sarcopenia in Older People	60-74 = 12,5 % ≥75 = 55 %	Sarcopenia 4,0 (0,4) No sarcopenia 4,1 (0,2)
Gariballa, *et al.*, 2018 ^17^	432	Sarcopenia 79 (7) No sarcopenia 77 (6)	_____	Longitudinal prospectivo	European Working Group on Sarcopenia in Older People	10 %	Sarcopenia 35,5 (5) No sarcopenia 38,1 (5)
Yoo, *et al.* 2018 ^18^	327	77,76 (9,7)	Hombres 78 (23,9); Mujeres 249 (76,1)	Longitudinal retrospectivo	Asian Working Group for Sarcopenia	37,3 %	Sarcopenia Hombre 3,68 (0,4) Sarcopenia Mujer 3,76 (0,44)
Zhou, *et al.*, 2017 ^19^	240	73 (7)	Hombres 190 (79,2); Mujeres 50 (20,8)	Longitudinal prospectivo	Tomografia axial	28,8 %	Sarcopenia 35,22 (4,12) No sarcopenia 37,31 (4,31)
Harimoto, *et al.*, 2016 ^20^	296	<70 hombres 123 <70 mujeres 34 ≥70 hombres 98 ≥70 mujeres 41	_____	Longitudinal retrospectivo	Tomografia axial	37,8 %	< 70 anos 4,0 (0,5) ≥ 70 anos 3,9 (0,5)
Hirasawa, *et al.* 2016 ^21^	136	68,6	Hombres (82,4)	Longitudinal retrospectivo	Tomografia axial	47,8 %	Sarcopenia 3,8 (0,006) No sarcopenia 4,1 (0,05)
Ishihara, *et al.*, 2016 ^22^	71	Sarcopenia 65,5 No sarcopenia 61	Hombres 50 (70,4) Mujeres 21 (29,6)	Longitudinal prospectivo	Tomografia axial	63,4 %	Sarcopenia 3,90 (4,0) No sarcopenia 4,38 (4,4)
Sugimotoa, *et al*., 2016 ^23^	418	77,3 (7,0)	Hombres 139 (33,3); Mujeres 279 (66,8)	Longitudinal retrospectivo	European Working Group on Sarcopenia in Older People	21,1 %	Sarcopenia 4,4 (0,3) No sarcopenia 4,4 (0,3)
Wang, *et al*., 2016 ^24^	255	65,14 (10,81)	Hombres 190 (74,5) Mujeres 65 (25,5)	Longitudinal prospectivo	European Working Group on Sarcopenia in Older People	12,5 %	Sarcopenia 34,01 (4,10) No sarcopenia 38,26 (4,02)
Kim, *et al*., 2015 ^25^	538	No sarcopenia 78,0 (2,6); Sarcopenia 78,5 (2,4)	Mujeres (100)	Longitudinal prospectivo	European Working Group on Sarcopenia in Older People	39,6 %	General 4,27 (0,22)
Gariball, *et al*. 2013 ^26^	432	Sarcopenia 79 (7,0); No sarcopenia 77 (6,0)	_____	Longitudinal prospectivo	European Working Group on Sarcopenia in Older People	10 %	Sarcopenia 35,5 (5) No sarcopenia 38 (5)
Harimoto, *et al*., 2013 ^27^	186	Sarcopenia 67 (11) No sarcopenia 66 (10)	_____	Longitudinal retrospectivo	Tomografia Axial	40,3 %	Sarcopenia 3,8 (0,4) No sarcopenia 4,0 (0,4)

En lo que respecta al diseño del estudio, se verificó que siete eran longitudinales prospectivos y, el resto, retrospectivos. Entre los 14 estudios, se alcanzó una muestra de 4.071 participantes, la más pequeña con 71 (22) y la mayor con 538 (25). Además, se encontró que la menor media de edad fue de 65,14 (24) y, la mayor, de 83,34 ^15^.

Los estudios se hicieron en adultos mayores ubicados en diferentes lugares: 11 en servicios de hospitalización, dos en el domicilio y uno en un consultorio de geriatría. Además, seis estudios se hicieron en adultos mayores con diagnóstico de cáncer, seis en personas evaluadas de rutina, uno estaba en el periodo posoperatorio de una fractura y otro tenía diagnóstico médico de demencia.

La detección de la sarcopenia en los 14 estudios de la revisión respondió a diversos criterios diagnósticos: en siete estudios, se usaron los criterios del *European Working Group on Sarcopenia in Older People; en* cinco, la tomografía computarizada y, en *dos, los criterios del Asian Working Group for Sarcopenia.*

La prevalencia de sarcopenia en el adulto mayor varió entre el 10 ^17^ y el 72,02 % ^15^. Además, algunos estudios caracterizaron la sarcopenia según el sexo. En el estudio de Gariballa, *et al.*^17^, se estableció que 29 (66 %) hombres y 176 (45 %) mujeres presentaban sarcopenia. En la otra publicación de Gariballa, *et al.*^26^, se detectó en 29 (66 %) mujeres . Además, Harimoto, *et al.*^27^, determinaron que 50 hombres y 25 mujeres presentaban sarcopenia (cuadro 2).

Los autores de los artículos propusieron diferentes análisis estadísticos para asociar la sarcopenia con la albúmina (cuadro 3). Algunos utilizaron la prueba t de Student o la de ji al cuadrado; otros emplearon la regresión lineal para determinar los coeficientes de asociación, así como la regresión logística para obtener el *Odds Ratio* (OR) y el *Hazard Ratio* (HR)

**Cuadro 3:** Tipo de análisis para establecer la asociación de sarcopenia y albúmina

Autor/año	Instrumento de diagnóstico de sarcopenia	Resultado	p
Regresión logística (OR)			
Kim, et al., 2015 ^25^	Indicador de sarcopenia	0,84 IC: 0,76;0,94	0,05
Regresión logística (HR)			
Harimoto, et al., 2013 ^27^	Tomografia axial	0,47 IC: 0,21;1,14	0,092
Regresión lineal			
Abbas, et al., 2020 ^14^	EWGSOP	-0,07 (-0,179 -0,046	0,244
Prueba t de Student			
Gong, et al., 2019 ^15^	EWGSOP	-	0,05
D'Alessandro, et al., 2018 ^16^	EWGSOP	4,0 (0,4)	0,21
Gariballa, et al., 2018 ^17^	EWGSOP	35,5 (5)	0,05
Yoo, et al., 2018 ^18^	AWGS - Hombres	3,68 (0,4)	0,295
	- Mujeres	3,76 (0,44)	0,084
Harimoto, et al., 2016 ^20^	Tomografía axial	3,8 (0,4)	0,9717
Sugimotoa Sugimoto, et al., 2016 23 EWGSOP		4,4 (0,3)	0,137
Gariballa, et al., 2013 ^26^	EWGSOP	35,5 (5)	0,05
Ji al cuadrado			
Zhou, et al., 2017 ^19^	Tomografía axial	35,22 (4,12)	0,001
Hirasawa, et al., 2016 ^21^	Tomografía axial	3,8 (0,06)	<0,001
Ishihara, et al., 2016 ^22^	Tomografía axial	3,90 (4,0;5,0)	<0,0001
Wang, et al., 2016 ^24^	EWGSOP	34,01 (4,10)	<0,0001

EWGSOP: *European Working Group on Sarcopenia in Older People;* AWGS: *Asian Working Group on Sarcopenia*

En la evaluación de la calidad metodológica con el instrumento MINORS, la suma de las categorías de todos los estudios dio un total de 11,21 puntos. Los ítems con menor puntuación fueron la pérdida en el seguimiento, con <5 %, la recolección prospectiva de los datos y el cálculo prospectivo del tamaño del estudio.

**Figura 2:**
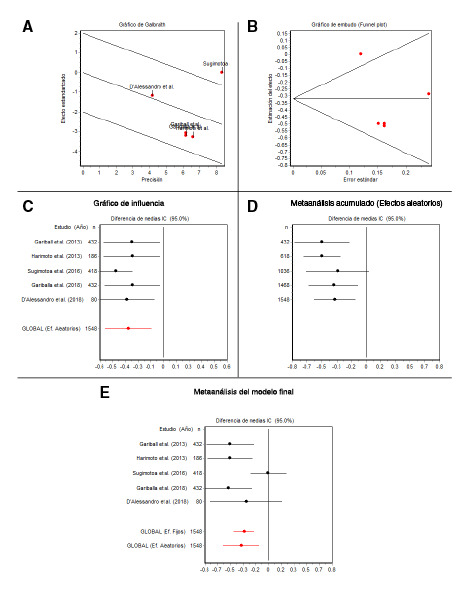
Metaanálisis de sarcopenia y albúmina sanguínea

En cuanto a la sarcopenia, se confirmó una elevada dispersión entre los estudios incluidos, como se observa en la figura 2A, en la prueba de Galbraith y el valor estadístico Q, con p=0,0233. Asimismo, se observó la ausencia de sesgos de publicación sobre el tema de estudio mediante el gráfico de embudo de la figura 2B y la prueba de Begg, con p=0,8065.

En el análisis de sensibilidad, se observó que los intervalos de confianza de las diferencias del puntaje promedio entre presencia o ausencia de sarcopenia y los valores de albúmina, fueron diferentes a cero (figura 2C). En el análisis que se presenta en la figura 2D, se evidencia que la adición sucesiva de las diferencias en los estudios no afectó el resultado final porque seguían siendo estadísticamente significativas.

En el metaanálisis final de los estudios, se observó que, entre las diferencias según la sarcopenia, el puntaje de la albúmina sanguínea fue estadísticamente mayor en los participantes que no la tenían frente a los que sí la presentaban, con excepción del estudio de Sugimotoa, *et al.*^23^, en el que fueron iguales en el modelo de efectos aleatorios (figura 2E).

En los estudios individuales, las diferencias por sarcopenia registraron puntajes de albúmina sanguínea favorables para quienes no tenían sarcopenia; asimismo, en la medida global, se evidenciaron mayores puntajes entre quienes no presentaban sarcopenia, con una diferencia de -0,3530 (IC95% -0,5900 a -0,1160).

Los resultados de esta revisión y metaanálisis muestran que los mayores valores de albúmina sérica se asociaron significativamente con la ausencia de sarcopenia en los adultos mayores. Se observó que la clasificación de la sarcopenia con base en la albúmina fue significativa en todos los estudios analizados. Sin embargo, no se estableció una asociación entre ambas variables de estudio.

## Mecanismo y prevalencia de la sarcopenia en el adulto mayor

En el estudio se determinó que la prevalencia de sarcopenia varió según la población de estudio y los factores asociados con ella. La sarcopenia implica una disminución progresiva de masa y fuerza o función del músculo esquelético con la edad, lo que implica un mayor riesgo de resultados adversos en salud. Existen procesos que contribuyen a la atrofia muscular y que están relacionados con edad, estado hormonal alterado, inflamación crónica, desequilibrio de la oxidorreducción, pérdida de las neuronas motoras a, disfunción mitocondrial muscular, alteración de la autofagia de miocitos, apoptosis acelerada de los mionúcleos y deterioro de la función de las células satélite ^28^.

En el proceso de envejecimiento, se dan diferentes mecanismos de atrofia de la fibra muscular que se relacionan con el desequilibrio en la síntesis y descomposición de las proteínas musculares, la resistencia anabólica a señales ambientales fundamentales, como la actividad física y el estado nutricional, que regulan la homeostasis muscular diurna y subyacen a las perturbaciones catabólicas relacionadas con la edad en la proteostasis muscular ^29^. Además, existen diversas causas subyacentes, entre ellas, se destacan la iatrogénica, la nutricional, la inactividad física, las enfermedades endocrinas, las metabólicas, las neurológicas y las osteoarticulares, entre otras ^30^.

En cuanto al diagnóstico de sarcopenia, en los estudios analizados se emplearon diferentes métodos. En este sentido, el problema central es que en la actualidad no hay un método único para determinar con precisión la masa muscular ^30^. Las mediciones antropométricas de la pantorrilla, por ejemplo, requieren puntos de corte por edad, sexo y raza, y deben considerarse algunas circunstancias como la obesidad o la retención de líquidos, por lo cual no tienen un buen desempeño diagnóstico ^31^.

Otras aproximaciones más precisas, como la absorciometría de rayos X (DEXA), la resonancia magnética o la tomografía computarizada, requieren de equipo y personal especializado, por lo que se emplean más en estudios de investigación que en el diagnóstico de sarcopenia como procedimiento de rutina por su alto costo ^32^. La bioimpedancia eléctrica (BIA), por su parte, es menos precisa, y está influenciada por la temperatura corporal y el estado de hidratación, entre otros factores ^33^.

Frente a estas dificultades, la sarcopenia se diagnostica mediante la prueba funcional del músculo y la evaluación de la fuerza. Sin embargo, estos estudios también requieren algunos equipos, instrumentos para determinar la fuerza, así como áreas especiales y, además, existen diversos criterios y puntos de corte que deben ser validados para cada población de estudio ^30^.

En esta revisión se estableció que la proporción de adultos mayores considerados sarcopénicos varió entre 10 ^15^^,^^26^ y 72,02 % ^17^^)^ con los diferentes métodos de evaluación y que hubo una estratificación de la sarcopenia según la edad, con predominio en aquellos con edad igual o superior a 75 años ^16^. En varios estudios se demostró que, después de la octava década de la vida, las concentraciones de testosterona en los hombres disminuyen rápidamente, lo que puede contribuir a la pérdida de la masa corporal magra y al aumento de la sarcopenia ^34^^,^^35^.

La sarcopenia relacionada con la edad es un problema grave de salud mundial en los adultos mayores y tiene implicaciones para la comunidad, ya que lleva a la incapacidad, y constituye una carga económica significativa para la familia y los servicios de salud ^35^. Las evidencias muestran que, a medida que aumenta la edad, la frecuencia de sarcopenia también aumenta, especialmente después de los 80 años. Además de la edad, existen otros factores asociados que aumentan la sarcopenia, como el estilo de vida, los hábitos de ejercicio, la presencia de enfermedades y la disminución de las concentraciones hormonales ^36^.

## Sarcopenia y albúmina en el adulto mayor

Por otro lado, las evidencias sobre los biomarcadores para la sarcopenia son todavía escasas; se sabe que no hay un único biomarcador ideal, sino que se debería disponer de un panel de herramientas complementarias como las imágenes diagnósticas, los biomarcadores séricos y las pruebas funcionales para su diagnóstico ^37^.

En diversos estudios se ha demostrado la asociación entre la sarcopenia y la alteración de los marcadores biológicos de inflamación y coagulación, como la interleucina IL-1 y la IL-6, el factor de crecimiento similar a la insulina de tipo 1, la hormona del crecimiento, el factor de necrosis tumoral- α (TNF-α) y otros marcadores inflamatorios ^37^ que actúan como parte de un sistema metabólico mayor o son mediadores de inflamación ^38^.

También, se ha evidenciado que el fragmento C-terminal de la agrina (CAF) aumenta en pacientes con fractura de cadera cuando hay sarcopenia ^37^. Asimismo, se ha estudiado la creatinina como metabolito para estimar la masa osteomuscular; sin embargo, los resultados de la creatinina urinaria varían de 11 a 30 %, dependiendo del tipo de dieta ^39^.

En cuanto a la albúmina sérica como biomarcador de la sarcopenia, la evidencia aún no es concluyente. En algunos estudios se la relaciona con un bajo rendimiento físico y menor fuerza muscular o masa muscular en adultos mayores; sin embargo, en otros no se ha evidenciado esta asociación, por lo tanto, aún no se sabe claramente si es un marcador definitivo de la sarcopenia en esta población ^40^.

Cabe resaltar que, hasta la fecha, no se cuenta con un indicador único, práctico, económico y fiable para el diagnóstico de la sarcopenia. En este contexto, se evaluó el papel de la albúmina como posible biomarcador, considerando que es un indicador del balance del metabolismo proteico en personas sin infección aguda.

La albúmina es la proteína plasmática más abundante en el organismo y tiene la función de regular el paso de agua y solutos a través de los capilares, manteniendo la presión oncótica coloidal dentro del sistema vascular. Considerada una proteína plasmática de gran importancia en la evaluación del estado nutricional, su disminución altera la cicatrización de heridas, causa problemas inmunológicos y reduce la masa corporal magra ^41^.

Hasta donde se sabe, este sería el primer metaanálisis que analiza la asociación entre sarcopenia y albúmina sérica; solo se encontraron algunas revisiones sistemáticas como la de Cabrerizo, *et al.*^7^, en la que se evaluó como indicador del estado nutricional y predictor de mortalidad en los adultos mayores; los autores concluyeron que los adultos mayores saludables tenían valores normales de albúmina y que la presencia de hipoalbuminemia puede servir para el pronóstico de complicaciones, incapacidad funcional y mortalidad precoz ^6^.

En otro metaanálisis sobre biomarcadores en sangre asociados con el riesgo de desnutrición en adultos mayores, se concluyó que la albúmina es un biomarcador útil de la desnutrición, incluso en presencia de inflamación crónica debida al envejecimiento o la enfermedad. Sin embargo, en contextos específicos, como enfermedades agudas o sepsis, debe evaluarse un conjunto de biomarcadores, entre ellos la albúmina ^9^, ya que es una proteína de fase aguda negativa cuyos niveles se reducen como reacción a la inflamación aguda ^6^.

La asociación entre mayores niveles de albúmina y la ausencia de sarcopenia en adultos mayores podría explicarse porque el principal déficit de nutrientes en esta población es el de proteínas ^42^, así que, en ausencia de inflamación, la albúmina constituye un adecuado biomarcador del estado de las proteínas viscerales del cuerpo y, ya que es la principal proteína sintetizada en el hígado, reflejaría el balance entre la ingestión y el gasto proteico. Sin embargo, su larga vida media, entre 18 y 21 días, no refleja cambios agudos en el estado nutricional ^43^.

Entre las fortalezas del estudio cabe mencionar que la revisión sistemática cuenta con un metaanálisis y corrobora los hallazgos cualitativos. Además, la búsqueda de artículos se hizo en cuatro bases de datos que cubren gran parte de la literatura especializada mundial.

Entre las limitaciones del estudio puede mencionarse que algunos autores clasificaron la albúmina como normal o deficiente, por lo que dichos estudios no se incluyeron en el metaanálisis. Otra limitación fue que en los estudios se emplearon hasta tres métodos de diagnóstico de sarcopenia, por lo que la clasificación del evento de interés no fue uniforme en todos los estudios, aspecto que refleja la actual situación de múltiples criterios para establecer el diagnóstico de sarcopenia.

## Conclusiones

Los estudios incluidos en la presente revisión revelaron que existe una asociación significativa entre los niveles bajos de albúmina y la sarcopenia en adultos de 60 o más años en diferentes entornos de cuidado. A pesar de estos, hay poca evidencia de alta calidad y los métodos de diagnóstico de la sarcopenia no son uniformes. Es necesario, por lo tanto, hacer estudios encaminados a determinar la asociación entre los diferentes marcadores biológicos (incluida la albúmina) y la sarcopenia, así como estudios de validación de puntos de corte de dichos marcadores para estimar la sensibilidad y la especificidad de cada uno, y seleccionar los más adecuados para una detección temprana de la sarcopenia que garantice un tratamiento multidisciplinario más efectivo.
